# Kikuchi Fujimoto Lymphadenitis: A Rare Association With COVID-19 Vaccination

**DOI:** 10.7759/cureus.45979

**Published:** 2023-09-26

**Authors:** Priya R Nair, Nandkishor J Bankar, Amruta Choudhary, Deepti Shrivastava

**Affiliations:** 1 Obstetrics and Gynaecology, Datta Meghe Medical College, Datta Meghe Institute of Higher Education and Research, Wardha, IND; 2 Microbiology, Jawaharlal Nehru Medical College, Datta Meghe Institute of Higher Education and Research, Wardha, IND

**Keywords:** lymphoma, asia, viral lymphadenopathy, covid vaccine, necrotising lymphadenitis, autoimmune disorders, tuberculosis, kikuchi fujimoto lymphadenitis

## Abstract

Kikuchi-Fujimoto disease (KFD), also known as histiocytic necrotizing lymphadenitis, is an uncommon cause of protracted cervical lymphadenopathy in both children and adults. Although the majority of cases have been documented in Asia, this disease has been characterized globally since it was first identified in Japan in 1972. KFD's etiology is not entirely understood, although various theories have been postulated. Usually benign, Kikuchi-Fujimoto disease resolves within six months. KFD must be distinguished from other causes of chronic lymphadenopathy, such as lymphoma, inflammatory illnesses, autoimmune conditions, viral lymphadenopathy, and also from tuberculosis, especially in India, where it is still endemic.

Here, we present the case report of a healthcare worker with a known case of hypothyroidism and autoimmune skin disorder who presented with prolonged fever, joint pain, and generalized lymphadenopathy post-COVID vaccination and was diagnosed as having KFD on biopsy, which can be associated with a very unusual presentation of this disease.

## Introduction

Kikuchi-Fujimoto disease (KFD), often called necrotizing histiocytic lymphadenitis, is primarily a rare entity characterized by lymphadenopathy and fever. It was first found in 1972 in Japan, and around 41 cases were reported until 2021 [1-3]. Usually, KFD is a benign, self-resolving ailment. It is acknowledged to be rare in the United States, with the majority of instances occurring in Asia, even though cases have been reported across the globe in literature. KFD is more common in women aged 20 to 35 [4]. However, there have been many recent publications describing the diagnosis in younger pediatric patients. 

Lin et al. published an article detailing their experience in 2003; 61 cases of KFD were reported in Taiwan over 15 years, from 1986 to 2000 [5]. In 2007, Kucukardali et al. published a review of 15 years, and they discovered 330 instances of KFD (in both adults and children) globally. Only 22 out of those 330 cases (7%) were discovered in the United States; the bulk were discovered in Asian nations [6]. The most often afflicted lymph nodes are those in the jugular-carotid and cervical regions, and Kikuchi-Fujimoto disease typically presents with fever and lymphadenopathy [7-10]. Since KFD can mimic other ailments like lymphoma, viral etiologies, and autoimmune diseases, particularly systemic lupus erythematosus (SLE), it is crucial to distinguish KFD as a clinical identity. Based on a study, 30% of cases of KFD were incorrectly diagnosed as lymphoma, as happened in this case also [11]. Therefore, a correct diagnosis with histopathology and clinical features is important for a good outcome.

## Case presentation

Patient history and clinical presentation: A 34-year-old health care worker (obstetrician and gynecologist) in Central India presented with a low-grade fever for four weeks, joint pain, and cervical lymphadenopathy after two doses of the COVID vaccine (Covishield), which is a recombinant chimpanzee adenovirus vector that encodes the glycoprotein of the SARS-CoV-2 spike (S). The first dose was taken in February 2021, and the second dose was taken in April 2021. The patient had a known case of hypothyroidism for 17 years on Thyronorm 100 mcg and had an autoimmune disorder, Lichen Planus, for 15 years with occasional lesions on her hands, face, and back, for which she was under topical steroids.

Diagnostic workup: Initial laboratory investigations for pyrexia under evaluation (Malaria, Dengue, Widal test) were done, which were negative. An antinuclear antibody (ANA) profile was done, which was spiked positive (1:100). The patient was initially managed with non-steroidal anti-inflammatory drugs, and the fever subsided. The patient then developed a high-grade fever with chills the next day after the precautionary dose of the vaccine in January 2022. Fever profiles were again repeated with negative results. The list of investigations is given below in Table 1.

**Table 1:** List of investigations TSH: Thyroid stimulating hormone, ESR: Erythrocyte sedimentation rate, CRP: C-reactive protein, IgE: Immunoglobulin E, RA Factor: Rheumatoid arthritis factor, IgM: Immunoglobulin M, ELISA: Enzyme linked immunosorbent essay, ANA: Anti-nuclear antibody, IFA: Immunofluorescence, HbA1c: Glycated hemoglobin.

Investigations	Value	Reference Range
Serum TSH	0.11 mIU/ml	0.4-4.5 mIU/ml
Mantoux test	3 mm	0-15 mm
ESR	74 mm/hr	0-20 mm/hr
CRP	23 mg/l	< 0.3mg/l
Serum Ferritin	74.35 ng/ml	10-160 ng/ml
IgE	315 k kUI/l	<300 kUI/l
RA Factor	13.5 IU/ml	<15 IU/ml
IgM Scrub Typhus ELISA	Positive (0.859)	<0.5
Brucella IgM antibodies	Negative (0.06)	Negative- <0.8 Borderline- 0.8-1.1 Positive- >1.1
Blood Culture	No Growth	
Widal Tube Test	Negative	Negative/Positive
Malarial Antigen Rapid	Negative	Negative/Positive
Enterochek IgM	Negative	Negative/Positive
ANA Blot by IFA	Speckled Positive (1:100)	Weak Positive (1:100) Moderate Positive (1:320) Strong Positive (1:1000) Very Strong positive (1:3200)
Complement-3	195.2 mg/dl	90-180 mg/dl
HbA1c	5.4%	Normal- <5.6% Prediabetic: 5.7-6.4% Diabetes: >6.5%

**Figure 1:**
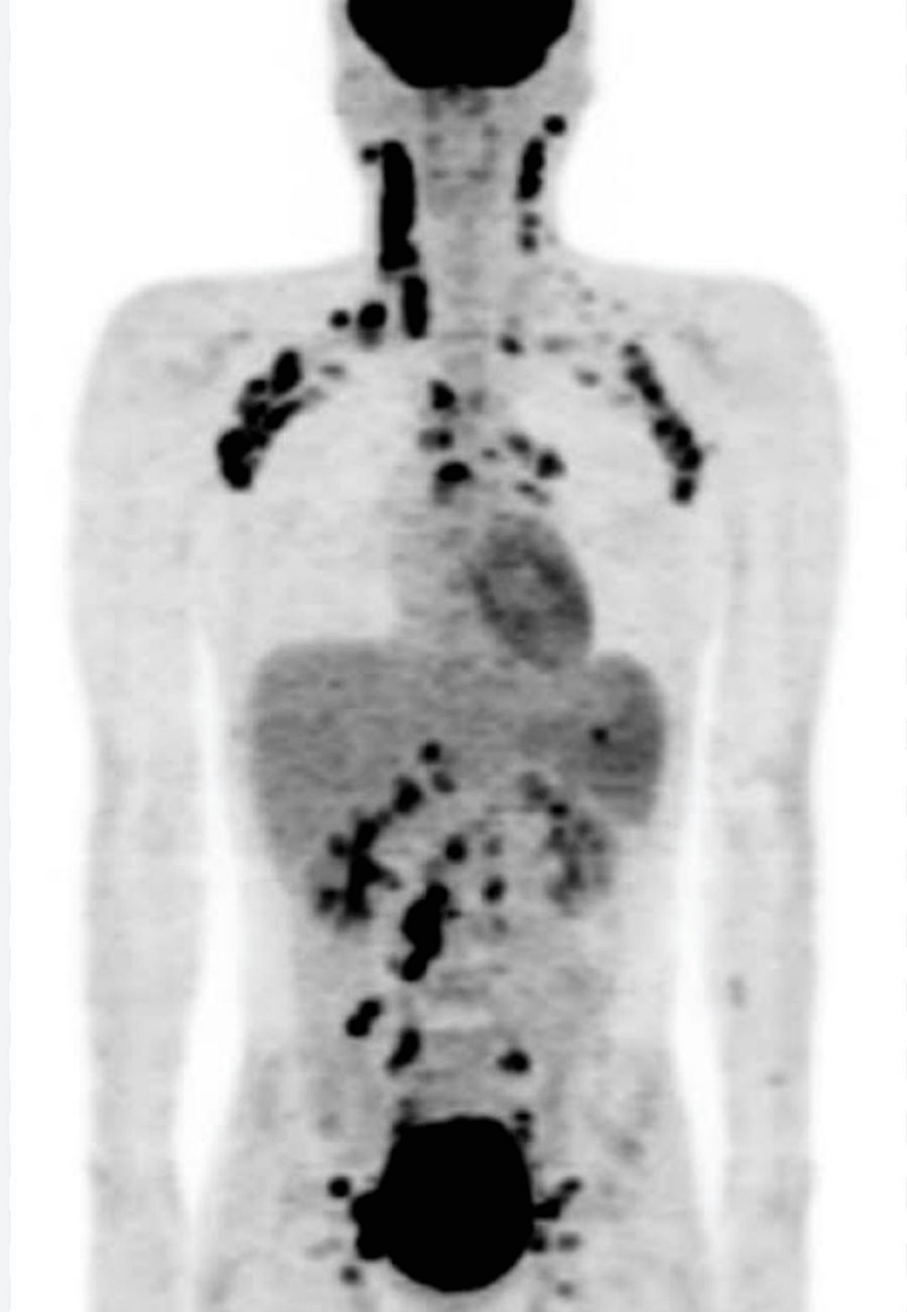
Whole-body FDG PET scan image FDG-PET: Fluorodeoxyglucose positron emission tomography

Ultrasonography of the abdomen and pelvis was normal, as was normal 2D echocardiography. Her thyroid profile was done, which showed features of hyperthyroidism, the dose of thyronorm was reduced from 100 to 75 mcg, and she received Doxycycline 100 mg two times a day and Azithromycin 500 mg once daily for a duration of seven days in view of a scrub typhus positive. She did not respond to the above treatment and was advised to be admitted. She was started on injectable antimalarial for three days, as India is endemic for malaria. She developed painful inguinal and cervical lymph nodes with severe joint pain. Her fluorodeoxyglucose positron emission tomography (FDG-PET) scan was suggestive of generalized lymphadenopathy with diffused increased metabolism in bilateral cervical and axillary, mediastinal, abdominal, and lymph nodes in the retroperitoneum with increased metabolism in liver and spleen, and the likely possibility of lymphoma over infective or inflammatory etiology as shown in Figure 1.

**Figure 2:**
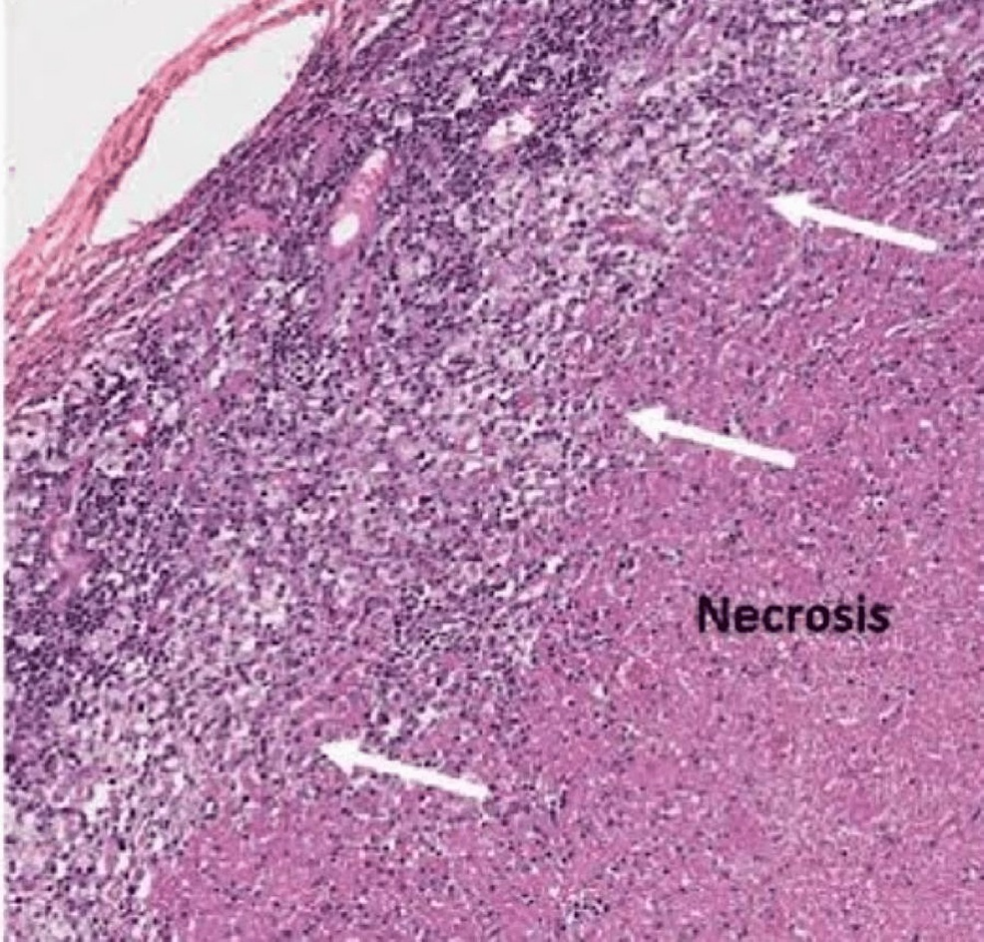
Histopathology report suggestive of Kikuchi Fujimoto lymphadenitis

A cervical lymph node biopsy was taken for confirmation of diagnosis and further management. Two cervical lymph nodes of approximate size (2x1.5 cm and 1x1 cm) were removed. The histopathology of the lymph nodes was suggestive of areas of infarctoid necrosis and karyorrhectic debris with a necrotic area surrounded by histiocytes and monocytoid cells, which was in favor of necrotizing histiocytic lymphadenitis, suggesting Kikuchi Fujimoto lymphadenitis, as shown in Figure 2. The patient was started on a low-dose steroid (Prednisolone) 60 mg in a tapering dose, starting with a three-times-a-day dose for seven days, followed by two times a day for another seven days, and once daily for the last seven days for a duration of 21 days, along with the anti-inflammatory drugs. The mainstay of treatment for this disease is non-steroidal anti-inflammatory drugs. Steroids are generally started in cases of generalized Kikuchi disease, central nervous system involvement, or severe extranodal involvement. The patient responded well to treatment, with no residual effect. Complications of KFD include meningoencephalitis, cerebellar ataxia, encephalitis, abscess of lymph nodes, acute renal failure, and hepatitis. Generally, KFD is self-limiting and resolves in 1-4 months with a relapse rate of 3%-4%. A one-year follow-up showed no relapse. Relapse is generally managed by corticosteroids, antipyretics, anti-inflammatory drugs, and, in some cases, hydroxychloroquine.

## Discussion

Although the exact origin of KFD is still unknown, infectious and immunological etiologies have been postulated [12]. This illness is believed to be an overreaction of the immune system to infections and chemical or physical agents. Brucella, Toxoplasmosis, Parvovirus B19, Bartonella, human herpes virus, cytomegalovirus, henslae, Epstein-Barr virus, Yersinia enterocolitica, parainfluenza, paramyxovirus, and human immunodeficiency virus are only a few of the known agents that may be present [12-15]. Serological and molecular research, however, has not been able to pinpoint a single particular pathogen. This means that rather than only relying on a physical examination and a patient's medical history, KFD has to be diagnosed using invasive techniques, e.g., excisional biopsy (for observing cellular alterations).

The Japanese people have the greatest rates of Kikuchi illness prevalence, although more recently, cases are being reported from across the world [16]. The current case has also been reported from India. Deaver et al. [17] stated that this disease's clinical course has both specific and non-specific features, with unilateral cervical lymphadenopathy serving as the latter. Although axillary and mediastinal lymph nodes are among the other groups that lymphadenopathy can affect, it frequently affects the cervical lymph nodes. Two more usual clinical signs include night sweats and an inexplicable fever. Our patient also stated all of the aforementioned usual symptoms. Less common problems include myalgia, headache, night sweats, tiredness, rash, arthralgia, weight loss, and stomach pain. Histopathological examination of a lymph node biopsy reveals a distorted architecture of lymph nodes, which are used to confirm the diagnosis. Necrotic nodules and nuclear fragmentation debris from cellular apoptosis are often seen [18]. Our patient's biopsy results were quite comparable; hence, KFD was determined to be the most likely diagnosis. KFD is frequently confused for tuberculosis, lymphoma, systemic lupus erythematosus, and even metastatic adenocarcinoma because of similar clinical features. Although it is uncommon, patients may exhibit peripheral and central nervous system involvement [19]. Though our patient did not exhibit central or peripheral nervous system involvement, she had the above-mentioned features. Therefore, when considering differential diagnosis, any doctor who encounters a case of lymphadenopathy should keep in mind KFD. KFD is self-limiting and resolves within 4-6 months without any residue. Symptomatic treatment consists of analgesics, antipyretics, and sometimes steroids.

## Conclusions

Kikuchi-Fujimoto disease is a rare entity that has a benign course without long-term residual effects. The most common presentations include fevers and lymphadenopathy, although there can be a variety of additional symptoms. KFD should be considered in the patient’s differential diagnosis when chronic lymphadenopathy is evident. Although the exact pathophysiology of this illness is unknown, it is speculated that its origin may be post-viral or connected to an autoimmune disorder, notably SLE. Thus, it should be given some thought to monitor patients and take an autoimmune workup into account. In cases of severe symptoms, corticosteroids may be considered a kind of treatment. Despite the fact that symptoms typically go away after six months, reports are linking KFD to bad outcomes.
